# Comorbidities and Quality of Life among Breast Cancer Survivors: A Prospective Study

**DOI:** 10.3390/jpm5030229

**Published:** 2015-06-26

**Authors:** Mei R. Fu, Deborah Axelrod, Amber A. Guth, Charles M. Cleland, Caitlin E. Ryan, Kristen R. Weaver, Jeanna M. Qiu, Robin Kleinman, Joan Scagliola, Joseph J. Palamar, Gail D’Eramo Melkus

**Affiliations:** 1 College of Nursing, New York University, New York, NY 10010, USA; E-Mails: chuck.cleland@nyu.edu (C.M.C.); cer365@nyu.edu (C.E.R.); qiujeanna@gmail.com (J.M.Q.); gdm3@nyu.edu (G.D.M.); 2 Department of Surgery, New York University School of Medicine, New York, NY 10016, USA; E-Mails: Deborah.Axelrod@nyumc.org (D.A.); amber.guth@nyumc.org (A.A.G.); 3 NYU Clinical Cancer Center, New York, NY 10016, USA; E-Mails: robin.kleinman@nyumc.org (R.K.); joan.scagliola@nyumc.org (J.S.); 4 Department of Population Health, New York University, New York, NY 10016, USA; E-Mail: joseph.palamar@nyu.edu

**Keywords:** comorbidity, breast cancer, quality of life, chronic illness

## Abstract

Many breast cancer survivors have coexistent chronic diseases or comorbidities at the time of their cancer diagnosis. The purpose of the study was to evaluate the association of comorbidities on breast cancer survivors’ quality of life. A prospective design was used to recruit 140 women before cancer surgery, 134 women completed the study. Comorbidities were assessed using self-report and verified by medical record review and the Charlson Comorbidity Index (CCI) before and 12-month after cancer surgery. Quality of life was evaluated using Short-Form Health Survey (SF-36 *v2*). Descriptive statistics, chi-square tests, *t*-tests, Fisher’s exact test, and correlations were performed for data analysis. A total of 28 comorbidities were identified. Among the 134 patients, 73.8% had at least one of the comorbidities, 54.7% had 2–4, and only 7.4% had 5–8. Comorbidities did not change at 12 months after surgery. Numbers of comorbidities by patients’ self-report and weighted categorization of comorbidities by CCI had a similar negative correlation with overall quality of life scores as well as domains of general health, physical functioning, bodily pain, and vitality. Comorbidities, specifically hypertension, arthritis, and diabetes, were associated with poorer quality of life in multiple domains among breast cancer survivors. Future research should consider the combined influence of comorbidity and cancer on patients’ quality of life.

## 1. Introduction

Breast cancer is the most commonly diagnosed cancer among women worldwide. Approximately one in eight women will be affected by breast cancer during her lifetime, and more than 2.9 million breast cancer survivors currently reside in the United States [1]. A woman who receives a breast cancer diagnosis may already have been diagnosed with or treated for a coexistent chronic disease, or commonly known as comorbidity [2]. The prevalence of comorbidities among women treated for breast cancer aged older than 66 is 32.2%, a statistic comparable to those without cancer at 31.8% [3]. Women with breast cancer have similar risks as those without cancer for developing chronic illnesses or comorbidities due to the natural effects of aging; however cancer survivors are at risk for chronic conditions (such as obesity, hypertension, diabetes, dyslipidemia and decreased bone mass) not only because of the natural aging process, but sometimes due to the late effects of cancer treatment [4].

The presence of comorbidities in patients with cancer has been negatively associated with patients’ health outcomes. Poorer survival from cancer has been found overall in cancer survivors with comorbidities compared to those without [5,6]. Breast cancer survivors with comorbidities are more likely to experience specific adverse outcomes such as delay and non-completion of radiation therapy [7], more likely to receive only breast conserving surgery without radiation therapy [8], and less likely to receive chemotherapy [9]. Breast cancer survivors with increased comorbidities have also been found more likely to experience negative in-hospital events, a conclusion reached after review of over 70,000 nationwide hospital discharges for patients undergoing breast cancer surgery [10]. Breast cancer survivors with increased comorbidities, as assessed by the Charlson Comorbidity Index (CCI) scores of 1, 2 and >3, had higher risks of non-routine disposition, prolonged hospitalization and in-patient death compared to breast cancer survivors with a CCI score of zero. Breast cancer survivors with comorbidities are therefore susceptible to negative outcomes along their cancer treatment continuum. The influence of such comorbidities upon breast cancer survivors’ quality of life, however, is an area less studied, and deserves a closer examination. In addition, with increased awareness of the importance of individualized patient-centered care as well as the increased rates and length of breast cancer survivals, quality of life becomes the focal parameter for breast cancer survivorship [1,2].

Research studies investigating the effects of comorbidities upon breast cancer survivors’ quality of life have been limited by cross-sectional study designs and the assessment of comorbidities by using sole patient self-report. Deshpance and colleagues [2] investigated the association of chronic disease burden and quality of life among a group of breast cancer survivors one year post-diagnosis. The researchers found breast cancer survivors with higher chronic disease burden (as measured by Katz’s measure of comorbidity), to report lower physical and social functioning (as assessed by the RAND 36-Item Health Survey). The majority of these patients, however, did not have a chronic disease burden (66% of the study population with a score of zero), and the assessment of comorbid illness was performed through sole patient self-report. Smith and colleagues [11] examined the relationship between cancer, comorbidities and health-related quality of life (HRQOL) in older cancer patients. The researchers found negative associations with physical and mental health in breast cancer survivors, especially in patients with two or more comorbidities who had been diagnosed with cancer within the past year. Although both of these studies highlight the negative effect of comorbidities upon breast cancer survivors’ quality of life, the need exists to prospectively evaluate the presence of medically diagnosed comorbidities before and after treatment, patterns of comorbidity development, as well as the influence of such comorbidities upon breast cancer survivors’ quality of life.

A global measure of comorbidity frequently employed by researchers is the Charlson Comorbidity Index [12,13]. CCI is a validated tool comprised of 17 comorbidities (4 atherosclerotic and 13 non-atherosclerotic), weighted according to disease severity, with a resultant score that can be used to predict treatment outcome and mortality risk. A limitation of the CCI, however, is that it does not capture all types of comorbidities that may prove relevant to health outcomes within the breast cancer patient population. For example, neither mental health comorbidities nor musculoskeletal comorbidities are captured within the CCI, although disease entities such as depression and arthritis frequently exist within the breast cancer patient population, and could seemingly affect health outcomes and quality of life.

In addition to the type of comorbidity index utilized, methodologies differ between studies in the assessment of patient comorbidities. Some studies rely upon sole patient self-report to evaluate comorbidities, whereas others utilize ICD-9 codes that reflect provider given diagnoses of that medical condition. The types of comorbidities evaluated in breast cancer survivors and the methods in which they are assessed, are therefore crucial to delineate in an investigation of the effects of comorbidities upon breast cancer survivors’ quality of life. The validity of comorbidity assessment must be verified and relevant comorbidities must be captured, in order to elicit the true influence of comorbidities within a patient population and detect pertinent patterns.

Numerous investigations have evaluated the influence of comorbidities upon breast cancer survivors’ outcome [13], but few have evaluated the influence of comorbidities upon breast cancer survivors’ quality of life. The purpose of the study was to evaluate the influence of comorbidities on breast cancer survivors’ quality of life. The specific aims were: (1) Explore patterns and changes of comorbidities prior to and at 12-month after cancer surgery; (2) Explore numbers and patterns of comorbidities evaluated through self-report and verified by medical record review in comparison with comorbidities assessed by the Charlson Comorbidity Index; and (3) Evaluate the associations of comorbidities with breast cancer survivors’ quality of life at 12 months after surgery.

## 2. Methods

### 2.1. Research Design and Participants

This study was approved by the institutional review board at New York University Langone Medical Center. A prospective design was used to recruit women who were over the age of 21, first time diagnosis of breast cancer (Stage I-III), and scheduled for surgical treatment including lumpectomy or mastectomy, sentinel lymph node biopsy or axillary lymph node dissection. Women with metastatic cancer (Stage IV) or prior history of breast cancer were excluded, since trajectory of cancer treatment is different [14]. Between April 2010 and June 2012, we prospectively enrolled 140 women and followed the participants for 12 months after surgery. Comorbidity in this study was defined as an existing diagnosis of a chronic illness prior to breast cancer treatment [10]. Comorbidities were assessed before cancer surgery and 12 months after surgery. Patterns of comorbidities were conceptualized as either an increase or decrease in numbers of comorbidities for each individual patient at 12 months after surgery [10]. Quality of life was evaluated using Short-Form Health Survey 36 Version 2 (SF-36 *v2*) only at 12 months after surgery. Among 134 participants who completed the study, 4 patients did not provide data on quality of life, therefore the 4 patients were excluded in the data analysis regarding quality of life. We stratified the participants into women who had at least one or more comorbidities and those who had none since the average weighted categorization of comorbidities calculated by Charlson Comorbidity Index (CCI) score was 1.59 (Mean = 1.59, SD = 1.66), whereas the average numbers of comorbidities reported by study participants and verified by medical record review was also 1.59 (Mean = 1.59, SD = 1.47).

### 2.2. Procedures

*Recruitment*: After the institutional review board approved the study, we used the successful procedures of recruiting and consenting participants used by the team in the previous studies [15,16]. Successful strategies included the use of *Invitation Flyer* that described the study. The *Invitation Flyer* was posted on the bulletin boards or breast cancer support website at the cancer center, and was also available in the reception areas of the center, examination rooms, and rooms holding support group meetings. In addition, healthcare providers such as nurses, oncologists, breast surgeons, and oncology radiologists at the cancer center referred women meeting the inclusion criteria to the study by distributing *Invitation Flyer* that described the study to the potential participants.

*Consent and Data Collection Process*: After reading the flyer, if a woman was interested in participating in the study, she would schedule a meeting with the researchers at that time or at other convenient time for them. During the meeting, the researchers confirmed her interest, determined if the woman is eligible for the study and the researchers again explained the study in detail and provide enough time for the woman to ask questions. If the woman agreed to participate, she would sign the consent form. All data collection was completed in person.

### 2.3. Instruments

*Demographic and Medical Information*: A structured interview tool [15,16] was used to gather demographic, medical, and clinical information regarding breast cancer diagnosis, stage of disease, type of adjuvant therapy, and treatment complications.

*Comorbidity Status*: Two approaches were designed to elicit broader and more detailed information and to ensure reliability of the women’s report regarding comorbidity status. First, participants responded to two open-ended interview questions: (1) In addition to breast cancer, what other illnesses or diseases do you have? (2) What medications do you take? Secondly, the researchers verified and validated the listed comorbidities by reviewing patients’ medical records before and 12 months after surgery. Participants were categorized as patients with or without comorbidities if self-reports were consistent with the medical records. To avoid overestimation, data from medical records were used to resolve any discrepancies between self-report, medications, and medical records.

*The Charlson Comorbidity Index*: A weighted index to evaluate risk of death from comorbid disease [12]. This system accounts for prognostic differences between disease entities by weighting comorbidities 1, 2, 3, or 6 according to the following categorizations: Weighted Score of 1: Myocardial infarct, congestive heart failure, peripheral vascular disease, cerebrovascular disease, dementia, chronic pulmonary disease, connective tissue disease, ulcer disease, mild liver disease or diabetes. Weighted score of 2: Hemiplegia, moderate or severe renal disease, diabetes with end organ damage, any tumor, leukemia or lymphoma. Weighted score of 3: Moderate or severe liver disease. Weighted score of 6: Metastatic solid tumor or AIDS. After calculating the weighted total of comorbidities, the score is placed into one the following indices: “0”, “1–2”, “3–4” or “>5”. With each increase in the comorbidity index there is a step-wise increase in observed mortality.

*The Short-Form Health Survey (SF-36) version 2 (SF-36v2)*: A short-form health survey of 36 questions with five -level responses. It yields an 8-scale profile of functional health and well-being scores as well as psychometrically-based physical and mental health summary measures and a preference-based health utility index. A higher score indicates better overall quality of life and each domain of quality of life. Reliability and validity of the instrument has been established with the median reliability coefficients for each of the eight scales equal to or greater than 0.80 [17]. SF-36v2 has been used to assess quality of life among breast cancer survivors [18].

### 2.4. Data Analysis

Characteristics of the participants were summarized using descriptive statistics (means, standard deviations for continuous variables and frequency distributions and proportions for qualitative variables). Distributions of baseline patient demographic and clinical characteristics were compared for patients with and without comorbidities using Chi-Squared tests for contingency tables and one-way analysis of variance for continuous variables. Correlations and t-tests, without assuming equal variance, were performed to examine associations between comorbidities and quality of life. All statistical tests were conducted at the 0.05 significance level (2-sided).

## 3. Results

### 3.1. Participants

One hundred and forty women were recruited before breast cancer surgery, 134 women completing the study with a 4% attrition rate. The reasons stated by those who did not complete the 12-month follow-up included: (a) significant travel distance (three patients); (b) death from cardiac related event (one patient); and (c) withdrawal of consent due to stress from chemotherapy (two patients).The mean age of participants was 56 years (range from 25–84 years), with the majority being married (53%) and had a bachelor (36.6%) or master or doctoral degree (30.6%). The majority of the study participants described their ethnicity as white (71.6%), with the remaining participants classifying their ethnicity as Asian (13.4%), African American (9.7%) or Hispanic (4.5%).

Participants had the following surgical procedures for breast cancer: sentinel lymph node biopsy (41.8%), axillary lymph node dissection (58.2%), mastectomy without construction (14.9%), mastectomy with immediate reconstruction (41.8%), and lumpectomy (43.3%). Participants also had the following adjuvant treatments: radiation (67.9%), chemotherapy (56.7%), radiation and chemotherapy (23.7%). There was no significant difference between participants with comorbidities and those without them in terms of cancer treatment (Table 1).

**Table 1:** Demographic and Clinical Characteristics of Participants.

	Total N = 134	No Comorbidity n = 35	With Comorbidities n = 99	*p* Value *
	Mean (SD; Range)	Mean (SD; Range)	Mean (SD; Range)	
**Age at Diagnosis (in years)**	56.2 (11.9; 25–84)	49.0 (9.1; 25–67)	58.8 (11.8; 28–84)	0.00 **
**Body Weight (in pounds)**				
Before Surgery	154.6 (36.8; 104.7–278.6)	149.1 (38.3; 104.7–278.6 )	157.0 (36.2; 106.0–277.3)	0.30
At 12 Months	154.8 (36.1; 104.9–284.4 )	147.6 (37.2; 106.8–284.4)	157.0 (35.6; 104.9–281.8 )	0.24
**Body Mass Index (BMI)**				
Before Surgery	26.4 (5.6; 17.7–46.1)	25.1 (5.6; 17.7–40.0 )	26.9 (5.5; 17.1–46.1 )	0.10
At 12 months	26.4 (5.5; 17.1–46.9)	25.0 (5.2; 17.8–39.7 )	26.9 (5.6; 17.1–46.9 )	0.08
**Highest Level of Education**	**n (%)**	**n (%)**	**n (%)**	0.33
High School or Below	38 (28.4)	6 (17.1)	32 (32.3)	
Associate’s Degree	6 (4.5)	1 (2.9)	5 (5.1)	
Bachelor’s Degree	49 (36.6)	14 (40.0)	35 (35.4)	
Master’s Degree	29 (21.6)	9 (25.7)	20 (20.2)	
Doctoral Degree	12 (9.0)	5 (14.3)	7 (7.1)	
**Marital Status**				0.57
Married	71 (53.0)	19 (54.3)	52 (52.5)	
Partnered	6 (4.5)	2 (5.7)	4 (4.0)	
Divorced/Separated	18 (13.4)	5 (14.3)	13 (13.1)	
Widowed	13 (9.7)	1 (2.9)	12 (12.1)	
Single or Never Partnered	26 (19.4)	8 (22.9)	18 (18.1)	
**Ethnicity**				0.04 *
Asian	18 (13.4)	7 (20.0)	11 (11.1)	
African American or Black	13 (9.7)	0 (0.0)	13 (13.1)	
White	96 (71.6)	25 (71.4)	71 (71.7)	
Hispanic/Latino	6 (4.5)	3 (8.6)	3 (3.0)	
Other	1 (0.7)	0 (0.0)	1 (1.0)	
**Employment Status**				<0.01 *
Unemployed	44 (32.8)	4 (11.4)	40 (40.4)	
Employed	90 (67.2)	31 (88.61)	59 (59.6)	
**Income**				0.16
More than enough	50 (37.3)	18 (51.4)	32 (32.3)	
Enough	61 (45.5)	14 (40.0)	47 (47.5)	
Not Enough	23 (17.1)	3 (8.6)	19 (19.2)	
N/A	1 (0.7)	0 (0.0)	1 (1.0)	
**Surgery**	**n (%)**	**n (%)**	**n (%)**	0.46
Mastectomy	20 (14.9)	7 (20.0)	13 (13.1)	
Lumpectomy	58 (43.3)	16 (45.7)	42 (42.4)	
Immediate Reconstruction	56 (41.8)	12 (34.3)	44 (44.4)	
**Radiotherapy**	91 (67.9)	23 (65.7)	68 (70.1)	0.67
**Chemotherapy**	76 (56.7)	21 (60.0)	55 (56.1)	0.84
**SLNB *vs.* ALND**				
SLNB	56 (41.8)	16 (45.7)	40 (40.4)	0.69
ALND	78 (58.2)	19 (54.3)	59 (59.6)	

Note: One-way analysis of variance was used for continuous variables and Chi-square tests for contingency tables. * means *p* < 0.05; ** means *p* < 0.01).

Participants with comorbidities were older, had a trend of high BMI at 12-month after cancer surgery. More participants with comorbidities were unemployed. More African American/black participants had comorbidities (Table 1).

### 3.2. Patterns and Changes of Comorbidities

The accordance of comorbidities between patient self-report verified by medical record review was 99%. Only one participant did not report depression when a diagnosis was documented in the medical record. A total of 28 comorbidities were identified through patient self-report verified by medical record review. Among the 134 patients, 73.8% had at least one of the comorbidities, 54.7% had 2–4, and only 7.4% had 5–8. The five most prevalent comorbidities in this patient population were as follows: hypertension (32.8%), arthritis (32.8%), thyroid problem (22.4%) hypercholesterolemia (12.7%) and diabetes (12.0%), see Table 2. Comorbidities assessed through self-report and verified by medical record review did not change during the 12 months after surgery.

**Table 2:** Comorbidities: Self-Report Verified by Medical Record Review.

N = 134	Comorbidities Assessed by Self-Report and Verified by Medical Record Review	Comorbidities Assessed by the Charlson Comorbidity Index	Participants with Comorbidities n (%)
1	Hypertension	*No*	44 (32.8)
2	Arthritis	*No*	44 (32.8)
3	Diabetes	Yes	16 (12.0)
4	Kidney Problems	Yes	4 (3.0)
5	Deep Vein Thrombosis	Yes	2 (1.5)
6	Vascular & Venous Problems	Yes	5 (3.7)
7	Heart Disease	Yes	11 (8.2)
8	Thyroid Problem	*No*	30 (22.4)
9	Hypercholesterolemia & Hyperlipidemia	*No*	17 (12.7)
10	GI disorders or GERD	Yes	12 (9.0)
11	Asthma	*No*	9 (6.7)
12	Depression	*No*	5 (3.7)
13	Anxiety	*No*	3 (2.2)
14	Secondary Cancers	Yes	6 (4.5)
15	Anemia	Yes	1 (0.7)
16	Multiple Sclerosis	*No*	1 (0.7)
17	Seizures	*No*	2 (1.5)
18	Hemangioma Liver	Yes	1 (0.7)
19	Autoimmune Lupus	*No*	1 (0.7)
20	Peripheral Neuropathy	*No*	1 (0.7)
21	Aneurysm	*No*	1 (0.7)
22	Myocardial Infarction	Yes	0 (0.0)
23	Cerebrovascular Disease	Yes	0 (0.0)
24	Connective Tissue Disease	Yes	0 (0.0)
25	Hemiplegia	Yes	0 (0.0)
26	Leukemia	Yes	0 (0.0)
27	Malignant Lymphoma	Yes	0 (0.0)
28	AIDS	Yes	0 (0.0)

Comorbidities through self-report and verified by medical record review *versus* assessed by the Charlson Comorbidity Index, did not differ significantly except one condition, arthritis. Arthritis, the equally most prevalent comorbidity as hypertension, affected more than one third of the study participants but is not assessed in calculating the Charlson Comorbidity Index. The other top comorbidities reported by study participants, hypertension, diabetes and heart disease, are conditions that are recognized by CCI, yet hypertension is not weighted for CCI calculation and hypercholesterolemia is not assessed by CCI. The average weighted categorization of comorbidities, calculated by Charlson Comorbidity Index (CCI) score was 1.59 (Mean = 1.59, SD = 1.47), whereas the average numbers of comorbidities reported by study participants and verified by medical record review was 1.59 (Mean = 1.59, SD = 1.66).

### 3.3. Comorbidities and Breast Cancer Survivors’ Quality of Life

Numbers of comorbidities, as assessed through self-report and verified by medical record review, was found to have a negative correlation with overall quality of life (r = −0.23, df = 129, *p* < 0.01). Likewise, weighted categorization of comorbidities, as conveyed by the Charlson Comorbidity Index score, was found to have a negative correlation with overall quality of life assessed by the SF-36v2 (r = −0.26, df = 129, *p* < 0.01).

Numbers of self-reported comorbidities verified by medical record review had negative correlations with the domain of physical functioning (r = −0.29, df = 128, *p* < 0.01), bodily pain (r = −0.23, df = 128; *p* < 0.01), vitality (r = −0.19, df = 129; *p* < 0.05), and social functioning (r = −0.19, df = 129; *p* < 0.05). BMI prior to surgery (r = 0.23, df = 127; *p* < 0.01) and age (r = −0.32, df = 128; *p* <0.01) had negative correlations with the domains of physical functioning.

In addition, certain individual comorbidities (*i.e*., hypertension, arthritis, and diabetes) were negatively associated with multiple domains of quality of life, including physical functioning, general health, bodily pain, and vitality. There were no significant associations between individual comorbidities and the domains of role-physical social functioning, and role-emotional emotional health (Table 3). Participants with arthritis, in comparison to those without it, had significantly lower scores in the domains of physical functioning (66.3 *vs.* 83.6), bodily pain (69.3 *vs.* 81.4), and general health (64.6 *vs.* 74.2). Participants with diabetes had significantly lower scores in physical functioning (62.2 *vs.* 80.0) and bodily pain (65.5 *vs*. 79.2) in comparison to those without diabetes. In addition, patients with hypertension were found to have significantly lower scores in physical functioning (71.4 *vs.* 81.2) in comparison to those without it. However, after adjustment for multiple testing, only the association between arthritis and physical functioning remained significant (*p* < 0.01).

## 4. Discussion

By utilizing a prospective design, assessing comorbidities before and after cancer surgery, looking for patterns of comorbidity, and using medical diagnoses to verify patient self-report, this investigation sought to reach beyond the scope of comorbidities assessed by the Charlson Comorbidity Index, and discover not only which comorbidities influenced breast cancer survivors’ quality of life, but also to quantify their affect. Many studies have demonstrated the detrimental influence that comorbidities have upon breast cancer survivors’ overall survival, and this investigation provides evidence as to the negative affect that comorbidities also exert upon breast cancer survivors’ quality of life.

**Table 3:** Quality of Life: SF36v2 Scores.

N = 130 ^#^	Hypertension		Arthritis		Diabetes		Heart Disease		Hypercholesterolemia & Hyperlipidemia	
	No n = 86	Yes n = 44	No n = 87	Yes n = 43	No n = 114	Yes n = 16	No n = 120	Yes n = 10	No n = 113	Yes n = 17
	Mean (SD)	Mean (SD)	Mean (SD)	Mean (SD)	Mean (SD)	Mean (SD)	Mean (SD)	Mean (SD)	Mean (SD)	Mean (SD)
**Physical Functioning**	81.2 (21.9)	71.4 (22.9) *	83.6 (18.9)	66.3 (25.2) ***	80.0 (20.9)	62.2 (28.6) *	79.2 (21.3)	62.0 (32.6)	76.8 (22.1)	84.7 (25.5)
**General Health**	73.1 (22.0)	67.5 (22.9)	74.2 (21.6)	64.6 (23.0) *	72.5 (21.9)	60.5 (23.7)	72.4 (21.9)	55.7 (22.8)	69.9 (22.4)	78.4 (22.1)
**Bodily Pain**	78.7 (23.7)	75.2 (22.7)	81.4 (21.3)	69.3 (25.5) ***	79.2 (22.8)	65.5 (24.3) *	78.7 (22.5)	63.0 (29.6)	76.9 (23.6)	81.7 (22.2)
**Vitality**	52.3 (17.1)	50.9 (16.5)	54.2 (15.5)	46.9 (18.7) *	52.6 (16.6)	46.3 (18.8)	52.5 (16.3)	44.6 (22.5)	51.1 (16.5)	56.5 (19.6)
**Social Functioning**	83.3 (26.6)	84.1 (21.6)	86.1 (23.1)	78.5 (27.9)	83.6 (25.1)	83.6 (24.5)	85.1 (23.9)	67.1 (30.8)	83.3 (25.0)	85.3 (25.1)
**Role-Physical**	78.6 (27.8)	80.9 (23.6)	82.3 (25.6)	73.6 (27.2)	80.6 (26.0)	70.7 (28.2)	79.7 (26.3)	76.1 (28.2)	79.9 (25.8)	75.4 (30.9)
**Role-Emotional**	86.2 (23.0)	85.6 (23.0)	88.2 (21.1)	81.6 (26.1)	86.3 (22.9)	84.4 (23.6)	86.9 (22.2)	76.5 (29.5)	85.8 (23.1)	87.8 (22.3)
**Emotional Health**	60.3 (15.8)	60.3 (15.6)	61.9 (14.4)	57.0 (17.8)	60.9 (15.3)	56.3 (18.7)	60.9 (15.4)	54.2 (18.5)	59.6 (15.6)	65.2 (15.9)

#: 4 patients did not provide data on quality of life. * *p* < 0.05 ** *p* < 0.01 *** *p* < 0.00.

Comorbidities were assessed in this study through patient self-report, and were verified through the review of medical records. A well-recognized limitation for prior investigations was the reliance upon patient self-report for the assessment of breast cancer survivors’ comorbidities. However, we found that patient self-report corroborated with medical diagnoses 99% of the time, providing evidence as to the reliability of patient self-report measures when assessing for comorbidities in this patient population. It should be noted that our study sample was highly educated with approximately 30% of the participants had advanced educational degrees. This may enhance their ability to report comorbidity conditions. A total of 28 comorbidities were identified during our assessment of breast cancer patients, with 78.1% of patients having at least one of the comorbidities, 54.7% having 2–4 and 7.4% having 5–8 comorbidities. Given the prevalence of comorbidities in this patient population, it is very relevant to assess for influence upon patients’ quality of life, as the majority of patients have been diagnosed with at least one of the comorbidities.

Two distinct measures were utilized in this investigation to explore associations among breast cancer survivors’ comorbidities and quality of life: The Charlson Comorbidity Index and the numerical tabulation of self-reported comorbidities that were verified by medical record review. These dual measures were employed to facilitate comparisons to other investigations and populations, as well as to capture comorbidities not included in the calculation of CCI scores. We found elevations in numbers of comorbidities compiled by the tabulation method and CCI scores to have similar, significant, negative correlations with patients’ overall quality of life (Pearson correlations of r = −0.23 and r = −0.26, respectively). Although these two measures differed in methodology and types of comorbidities assessed, they both revealed similar results; numbers of comorbidities by self-report and average weighted categorization of comorbidities by CCI had negative associations with overall quality of life among women treated for breast cancer 12 months after surgery.

Assessing comorbidities in this investigation yielded the following five most prevalent conditions: hypertension (32.8%), arthritis (32.8%), thyroid problem (22.4%) hypercholesterolemia (12.7%) and diabetes (12.0%). Although tabulation and CCI measures revealed overall similar results in that increased comorbidities are being negatively associated with quality of life, the tabulation method of comorbidities provided additional information. By assessing for types of comorbidities representative of this particular patient population of breast cancer survivors, we were better able to evaluate the association of comorbidities relevant to this patient population with quality of life For instance, over a third of our patients had comorbidities of arthritis and hypertension; such disease entities were not included in the calculation of Charlson Comorbidity Index scores, but potentially could have a negative influence on quality of life domains. For example, arthritis had negative associations with quality of life in several domains, including physical functioning, general health, bodily pain, and vitality. It should be noted the negative association between arthritis and physical functioning remains even after adjusting for multiple testing. This indicates arthritis could be an important comorbidity that influences patients’ quality of life among this population. Therefore, by first identifying comorbidities most relevant to this particular patient population, we could then ascertain the effects of those conditions upon breast cancer survivors’ quality of life.

Utilizing the SF-36v2, we were able to demonstrate that patients with arthritis had significantly lower scores in the quality of life domains of physical functioning, general health, bodily pain, and vitality. Patients with hypertension had significantly lower scores in the domain of physical functioning, whereas patients with diabetes had significantly lower scores in the domains of physical functioning and bodily pain. Not only were breast cancer survivors with an increased number of comorbidities found to experience poorer overall quality of life, but in stratifying by disease entity, we were better able to understand which domains of quality of life were negatively associated with specific comorbidities.

A few limitations of this study deserve mention. First, our patient population consisted primarily of white women, and therefore certain comorbidities may have been over or under-represented. Thus, our findings may not be generalizable to a more heterogeneous patient population. Nevertheless, our study also found that African American/black participants all had at least one or more comorbidities. Second, patients’ quality of life scores were not assessed pre-operatively due to the fear that asking quality of life at the time point of cancer diagnosis might induce unnecessary emotional distress from patients. However, we are aware the absence of assessments of quality of life at pre-surgery prevents an evaluation of treatment effects on quality of life. The modest sample size limited the ability of the study to explore the effects of comorbidities in the context of other determinants of quality of life using advanced regression models. Future research needs to examine the effects of comorbidities on quality of life using larger samples. Despite these limitations, this study utilized a prospective, longitudinal design, assessing comorbidities before and after surgery, and verifying patient self-report of comorbidities with medically given diagnoses. By assessing beyond the comorbidities included in the Charlson Comorbidity Index, we were able to identify comorbidities relevant to this particular patient population, and discern patterns of disease presentation. In addition to illustrating an overall negative correlation between an increased number of comorbidities and breast cancer survivors’ quality of life, we were able to stratify by disease entity and demonstrate how particular disease entities were associated with particular domains of patients’ quality of life.

## 5. Conclusions

Comorbidities are negatively associated with breast cancer survivors’ quality of life. Hypertension, arthritis, and diabetes have negative associations with multiple domains of breast cancer survivors’ quality of life. This study provides evidence as to the stability of comorbidity patterns within the breast cancer patient population, existing before and 12 months after surgery, and sheds light as to how such comorbidities are negatively associated with certain domains of patients’ quality of life. It is only after such knowledge is obtained regarding the relationships of comorbidities with breast cancer survivors’ quality of life that interventions can be designed to manage such comorbidities and optimize breast cancer survivors’ quality of life. Future research should consider larger and more diverse samples as well as the combined impact of comorbidity and cancer treatment upon breast cancer survivors’ quality of life.
